# Analysis of syntactic and semantic features for fine-grained event-spatial understanding in outbreak news reports

**DOI:** 10.1186/2041-1480-1-3

**Published:** 2010-03-31

**Authors:** Hutchatai Chanlekha, Nigel Collier

**Affiliations:** 1 National Institute of Informatics, Hitotsubashi 2-1-2, Chiyoda-ku, Tokyo, Japan

## Abstract

**Background:**

Previous studies have suggested that epidemiological reasoning needs a fine-grained modelling of events, especially their spatial and temporal attributes. While the temporal analysis of events has been intensively studied, far less attention has been paid to their spatial analysis. This article aims at filling the gap concerning automatic event-spatial attribute analysis in order to support health surveillance and epidemiological reasoning.

**Results:**

In this work, we propose a methodology that provides a detailed analysis on each event reported in news articles to recover the most specific locations where it occurs. Various features for recognizing spatial attributes of the events were studied and incorporated into the models which were trained by several machine learning techniques. The best performance for spatial attribute recognition is very promising; 85.9% F-score (86.75% precision/85.1% recall).

**Conclusions:**

We extended our work on event-spatial attribute recognition by focusing on machine learning techniques, which are CRF, SVM, and Decision tree. Our approach avoided the costly development of an external knowledge base by employing the feature sources that can be acquired locally from the analyzed document. The results showed that the CRF model performed the best. Our study indicated that the nearest location and previous event location are the most important features for the CRF and SVM model, while the location extracted from the verb's subject is the most important to the Decision tree model.

## Background

The spread of infectious diseases such as Avian H5N1 influenza and Pandemic A(H1N1) influenza, has drawn public concern for strengthening public health monitoring techniques for the speedy and precise detection of disease outbreaks. Nevertheless, the under-development of public health systems in many parts of the world has caused a significant barrier for developing an effective global indicator-based health surveillance system [1,2]. This situation has underscored the importance of report-based surveillance systems [1,3-7] as another crucial source of epidemic information to fill this gap.

One of the key information in which all of the report-based health surveillance systems are interested in is the place where the outbreak occurred, i.e. the spatial information of the outbreak events. However, for automatic encoding, systems tend to adopt ad-hoc strategies, generally in the form of detecting the first disease and location pair that match predefined criteria or similar heuristics. Although these strategies are effective for reducing the computational time and pruning out locations that are irrelevant to the outbreaks, they may lead to under-reporting of the outbreak or issuing reports at sub-optimal levels of granularity. Moreover, they also cause difficulties when extending the functionality of the health surveillance system to abstract the spatial attributes of every event reported in text, which is reported to be necessary for epidemiologic reasoning [8].

In order to tackle these limitations, a more sophisticated approach for analyzing the entire document is necessary. For the spatial attribute analysis, previous works on the spatial analysis of text have focused on, for example, location name identification [9,10], geographical grounding [11,12], extracting spatial attributes of specific, predefined events [3,13,14], and analysis of the geographic focus of web pages [15]. However, to our knowledge, less attention has been paid to event-spatial association.

The work presented in this paper builds on our previous studies [16]. Here, we extend our implementation of machine learning techniques for recognizing the spatial attributes of events. We also report an analysis of features' contribution and their combination to evaluate their impact on the recognition performance.

The remainder of this article is organized as follows. We first define the events considered in our work. Next, the details concerning our experimental data are explained. Then, the features and methodologies used for the spatial attribute annotation are discussed. In addition, the results of the experimentation are reported and analyzed. Finally, we discuss the limitations and problems with our methodologies and our future works. Note that most of the examples used for illustration were drawn from the BioCaster corpus [17].

### Event definition

In this work, events are considered predicates describing the state or circumstance in which something changes or holds true and which can be located in time. Linguistically, real-world events can be expressed through various grammatical constituents, such as using the noun phrase "Bird flu outbreak" to describe the sudden spread of bird flu, or using the verb phrase "report" to describe the act of communication or "is sick" to describe the state of people affected with a disease. We focus mainly on the events that are expressed through verb phrases; for example:

- Verbs, such as "die", "occur", and "spread"

- Copula verbs with adjectives, such as "is underway" and "was ill"

- To be + prepositional phrases, such as "is on board" or "is in progress", or "was in Indonesia".

In the following example, the expressions bold-marked represent an event as described above.

*Seventeen people have died and41 have been admitted to hospitals in Sichuan, China*, *suffering from an undiagnosed disease*.

### Difficulties in spatial attribute annotation

In the gold standard corpus development, we found that there are certain sets of event expressions that usually confuse human annotators when trying to select the geographical locations as the event's spatial attribute. The event expressions that usually caused disagreement are those that refer to background knowledge about the disease or hypothetical situation. For example:

*Human T-cell Lymphotropic Virus, Type1 (HTLV-1) occurs mostly in Japan, Caribbean countries and Africa. Doctors ****say****most people who contract it will showno symptoms*.

The events marked in boldface can be regarded as referring to generic knowledge. However, while one annotator considered these events as world knowledge, selecting the spatial attribute as "World", another annotator considered them as information about specific locations and selected Japan, Caribbean countries, and Africa as the spatial attribute.

### Impact of event class on spatial attribute annotation

As we mentioned earlier, there are some classes of event expressions that usually cause difficulties in spatial attribute annotation for human annotators. Therefore, we hypothesize that other spatially-irrelevant information, such as the class of event, may have an impact on the spatial attribute annotation. According to this observation, we decided to classify events according to the following spatial characteristics.

#### • Spatially locatable events

Events in this class truly occur in the world, and therefore, can be geographically located. For example:

*The ministry said the boy might have been infectedby sick chickens near his home*.

*Five days after returning to her hometown of Khon Kaen, she fell ill with Sars-like symptoms*.

Among the expressions that represent events in this class, there is a certain set of verbs that are often found in news reports. These verbs have a communicative function, and they are commonly referred to as reporting verbs [18]. Reporting verbs that refer to the same event are often scattered throughout a news story. In recognizing the locations of these verbs, it is important to collectively recognize these verb expressions as they refer to the same event; that is, all occur in the same place. Given this observation, we believe that it is advantageous to separate reporting events from other spatially locatable events. We decided to further classify spatially locatable events into two subclasses: 1) reporting events and 2) normal events.

#### • Generic information

Generic information is usually shown by non-eventive expressions, events that can not be positioned in space (or time), or generic events [19]. The following are examples of general information:

- General knowledge that is always true

- Imperative and interrogative sentences

- Sentences whose subjects are linked to their predicates (e.g., characteristics, attribute, etc.) via a copula verb

Examples of sentences that represent generic information are:

*The victim is a 12-year-old boy*.

*Enteric fever is caused by bacteria called Salmonella typhi, Salmonella paratyphi A, or Salmonella paratyphi B*.

#### • Hypothetical events

Hypothetical events are alternatives or occur in other possible worlds. Events in this group represent the perspective or anticipation of the speaker, or conditionally possible situations. The below sentence is an example of hypothetical events.

*If the virus mutates it could create a pandemic*

Since there is no off-the-shelf tool that is specifically tailored for this task, we decided to develop a system that is capable of classifying event expressions in terms of the above classes. For event classification, we used the Conditional Random Fields (CRF) [20] machine learning technique. To create the training corpus, human annotators were asked to classify a collection of event expressions into one of the four classes previously mentioned. The performance of the event classification based on n-fold cross validation strategy was 89.2% F-score. This was then used as input recognizing event classes for automatic spatial attribute annotation. Clearly, differences in performance across event classes make comparisons difficult between these classes but should allow direct comparisons to be made between spatial attribute features.

## Methods

### Experiment data

The corpus for the spatial attribute annotation task consisted of 100 news reports about disease outbreak events, randomly selected from the BioCaster gold standard corpus [17]. All of the news articles were manually marked-up with named entity tags [21], which includes diseases, viruses and person types. All the event expressions were pre-annotated by hand. The process used for this is as follows. All documents were shallow parsed for verb phrases. Then, we manually checked and marked-up the verb phrases that conformed to the event definition. There were 1326 clauses/sentences, with 1994 events in the experimental corpus.

In recognizing the spatial attribute of the events, apart from the named entity, other linguistic information, such as the grammatical dependency, or co-reference, might also be useful. To provide such information to the system, we passed the experimenting corpus through the pre-processing steps, which included:

1) dependency parsing [22]; to provide grammatical dependency information, such as the subject, object, etc., and

2) manual co-reference annotation; to identify the noun phrases, including pronoun, that are co-referents of the same real-world entity

Our corpus covered news articles published from 1996 to 2007. They reported outbreaks of 44 diseases in 45 countries. In some articles, one disease outbreak was reported in multiple countries. There are also some articles that reported the spread of multiple diseases within one country.

### Features for spatial attribute recognition

In order to develop an effective automatic system for recognizing a textual event's spatial attribute, the features to be used as the information source for the recognition task must be carefully selected. We asked 10 people, including the 2 linguists, 7 Ph.D. students in the Department of informatics at The Graduate University for Advanced Studies, and 1 post-doctoral researcher, about how they recognized the place where the event reported in the news occurred in order to gather their opinion. The results from our observation showed that they usually agreed on the following sources to be used for identifying the place of occurrence of the events. These sources are listed below. In the description of textual features, a "verb" means a verb phrase that represents the action or state. A "subject" means the subject of the verbal expression representing the action or state. An "object" refers to the direct or indirect object of the verbal expression representing the action or state.

**F1: **Location of subject

This feature represents the (possible) geographical location of the subject. They can be the location name in the subject modifier, the location name in the subject appositive, and the location name in the subject's relative clause among others.

**F2: **Location of subject's co-reference

This feature refers to the location names that appear in or modify the noun phrase that corefers to the same real-world entity as the subject of the in-focus verb.

**F3: **Location of object

This feature represents the (possible) geographical location of the object. It can be a location name in the object modifier, a location name in the object appositive, or a location name in the object's relative clause.

**F4: **Location of object's co-reference

This feature refers to the location names that appear in or modify the noun phrase that corefer to the same real-world entity as the object of the in-focus verb.

**F5: **Location of verb

We consider the location of the verb to be the location names that appear in the phrase that modifies directly the verb, such as the location name in prepositional phrases.

**F6: **Location of verb's coreference

In this work, verb coreference means a pair of verbs that have the same meaning (or the same sense) and the subjects of both verbs refer to the same real-world entity.

**F7: **Inference or co-reference of locations that appear in verb modifier

In some cases, there is no information about the event's geographical location appearing in the clause or sentence. However, human readers can infer the geographical location from the story.

**F8: **News agency location

The location of a news agency, especially the local news, can sometimes be used as a default location of the situation reported in the news article.

**F9: **Nearest location names

Previously mentioned location(s) that is closest to the event expression can usually be used as a clue for recognizing the actual location of that event. In this work, the nearest locations were considered according to the following heuristic:

For representing nearest location names, we created two sub-features, which are the country-level nearest location and the sub country-level nearest location. Both sub-features use the same heuristic for extraction.

**F10: **Location in news headline

Under certain circumstances, the location name appearing in the headline can be used as a default location for the situation reported in the news articles

**F11: **Previous verb's location

Without introducing an expression to indicate a change in geographical location, readers usually perceive the continually reported story as occurring in the same place. From this observation, the location recognized as the place where the event represented by textually-previous verbs occurred is introduced as another feature for spatial attribute recognition.

### Spatial attribute recognition approach

As discussed earlier, there is usually a certain set of textual features that human readers use for signaling the location of the events. To recognize the spatial attribute of an event, we formulate our task as 11-class categorization problem, i.e. selecting, among the feature sources that provide location information, the most reliable one that is likely to give the correct spatial attribute of the event.

In this experiment, we asked one of the annotators to enrich the annotation of each event expression in the corpus. Apart from annotating the spatial attribute, the annotator must select from the list of 11 signal features (cf. previous section) the one that is the most reliable source. The learning models were then on this data.

We employed various statistical machine learning techniques, such as CRF [20], Decision Tree [23], and Support Vector Machine (SVM) [24], to select the best feature to give the correct spatial attribute given a certain textual context. We used CRF++ [25] as a tool for CRF learning, libsvm [26] for SVM learning, and C4.5 [23] for Decision tree learning. The location extracted from the feature that the model selected is considered the event's spatial attribute predicted by the model. Parameter settings for each machine learning model are as follows:

- For CRF++, *f *and *c *parameters were set as 3 and 4, respectively.

- For SVM, linear kernel was used.

- For C4.5, the default setting was used.

As mentioned in the spatial attribute annotation task, there are certain sets of event expressions that usually cause confusion for human annotators in selecting the geographical locations as an event's spatial attribute. Based on this observation, apart from the 11 feature sources, information about an event's class and type of subject are also incorporated into the model in order to evaluate the relevance of this information for the spatial attribute annotation task.

The resulting feature set given to the learners is shown in Table 1.

**Table 1:** Feature encoding method for learning task

Feature	Value
11 location-related features	If geographical locations can be extracted from feature f_i_, then value of f_i _is encoded as "Y". Otherwise, value of f_i _is encoded as "N".
Event class	"Normal", "Report", "Information", "Hypothetical"
Subject type	"Disease_Germ" (disease or pathogen) "Symptom" (expression that indicates symptom of any disease) "Official" (a person who has the property of being an official) "Person" (a person who does not have the property of being an official) "Government Organization" "WHO" (World Health Organization) "WHO-related" (WHO agency or UN agency) "Organization" (other kinds of organizations) "Location" "Other" (none of above classes)

## Results

The results of attribute annotation are shown in Table 2. The scores for overall performance were based on micro-averaging. Note that the event class used as a feature in the model is the result from an automatic event classification system.

**Table 2:** Experimentation results for recognizing spatial attribute of events based on statistical machine learning approach

Features	Machine learning techniques	Event class				
		
		Normal	Reporting	Information	Hypothetical	Over all
11 location-related features	CRF	84.8 (85.8,83.8)	82.7 (81.9,83.5)	62.6 (63.0,62.2)	88.1 (92.5,84.1)	81.3 (81.9,80.7)
	SVM	84.6 (85.8,83.5)	83.7 (82.7,84.6)	54.8 (55.2,54.5)	81.0 (85.0,77.3)	80.0 (80.7,79.4)
	C4.5	78.8 (79.6,77.9)	85.7 (85.2,86.2)	44.3 (45.0,43.6)	64.4 (65.1,63.6)	74.9 (75.5,74.4)

11 location-related features + event class	CRF	**87.2** (88.3,86.1)	86.1 (85.6,86.6)	68.2 (68.4,67.9)	76.2 (80.0,72.7)	83.8 (84.5,83.1)
	SVM	86.6 (87.7,85.5)	83.7 (82.7,84.6)	65.8 (66.2,65.4)	78.6 (82.5,75.0)	82.6 (83.3,82.0)
	C4.5	82.8 (84.1,81.6)	**88.4** (87.9,88.9)	55.3 (56.8,53.8)	78.2 (79.1,77.3)	80.1 (81.0,79.2)

11 location-related features + subject type	CRF	85.7 (87.4,84.1)	85.8 (84.9,86.6)	75.5 (76.0,75.0)	80.95 (85.0,77.3)	84.1 (85.1,83.1)
	SVM	84.8 (86.4,83.2)	83.7 (82.7,84.6)	58.7 (59.1,58.3)	**83.3** (87.5,79.5)	80.8 (81.6,79.9)
	C4.5	79.5 (80.8,78.3)	87.2 (86.3,88.0)	61.3 (61.1,61.5)	61.2 (61.9,60.5)	78.0 (78.5,77.4)

11 location-related features + subject type + event class	CRF	86.7 (88.1,85.4)	87.1 (86.4,87.8)	**80.4** (80.6,80.1)	76.2 (80.0,72.7)	**85.5** (86.3,84.7)
	SVM	86.1 (87.8,84.4)	84.0 (83.1,84.4)	68.4 (68.8,67.9)	81.0 (85.0,77.3)	82.8 (83.7,82.0)
	C4.5	80.2 (80.9,79.5)	88.0 (87.5,88.5)	66.7 (68.7,64.7)	64.4 (65.1,63.6)	79.5 (80.1,78.8)

The attribute annotation results shown in this table were based on micro-averaging. The scores are shown in the form of "F-score (precision, recall)"

Comparing between the three machine learning techniques, CRF yielded the best performance (F = 87.2%). From the results, the CRF model, incorporating location-related features, subject type, and event class gained the highest performance.

While the CRF and SVM models with all features performed the best, incorporating only 11 location-related features and event class yielded a better result for Decision tree (F = 80.1%). Although the subject type feature obviously improved the performance for CRF (from F = 81.3% to 84.1%) and Decision tree model (from F = 74.9% to 78.0%), it has slight positive impact to the SVM model (from F = 80.0% to 80.8%). Nevertheless, the result from the models trained by the three machine learning techniques indicates that the event class and subject type features are useful for recognizing the spatial attribute of events in the information class.

In order to study the contribution of each spatial-related textual feature, another set of experiments was conducted. In these experiments, each spatial-related textual feature was removed from the model in an excluding-one-feature-per-training manner.

According to the results, compared to the model trained with all the features (cf. Table 2), removing the headline feature improved recognition performance for the SVM (from 82.8 to 83.3 F-score) and Decision tree models (from 80.1 to 80.95 F-score), while yielded the same level of performance for the CRF model. Excluding the feature from the event coreference also improved the performance of the SVM model, but degraded the performance of the Decision tree model. For the CRF model, removing the object/indirect object coreference feature raised the recognition performance to 85.9, compared to the 85.5 F-score in the model that was trained with all the features. In CRF and SVM models, the recognition performance was most reduced when excluding the previous event feature (F = 78.8% for CRF; F = 76.2% for SVM). On the other hand, the performance of the Decision tree model was the lowest when excluding the location-related subject feature (F = 73.9%).

We also analyzed spatial attribute recognition performance for each event class. For events in the normal class, removing the nearest locations and the previous event feature causes a significant drop in performance for the CRF model (F = 79.7% for CRF and 79.1% for SVM).

For events in the normal class, by removing the nearest locations and the previous event feature cause a significant drop in performance for the CRF model (F = 79.7% for CRF and 79.1% for SVM). Among the SVM models, removing the previous event features has the biggest impact on the spatial attribute recognition performance (F = 76.6%). Although the performance trend is quite similar between SVM and CRF models, the results from the decision tree model are obviously different. For the decision tree-trained model, removing the location-related subject feature has the most impact on the recognition performance (F = 72.8%).

For the spatial attribute recognition of reporting events, the results show that the performance was reduced the most when the spatial information of the subject and subject co-reference was removed from the model (F = 81.4% for CRF; F = 78.9% for SVM, and F = 78.7% for Decision tree). This situation is reflected in all the models trained by using the three techniques.

Next, we analyzed the spatial attribute recognition performance on the information class. For the CRF and SVM models, removing the previous event feature has the largest negative impact on recognition performance (F = 63.2% for CRF; F = 51.9% for SVM). While removing the object and indirect object coreference features has almost no impact on the CRF and SVM models in recognizing the spatial attribute of the information class, it surprisingly causes the biggest drop in performance for the decision tree model (F = 62.9%).

For the spatial attribute recognition of hypothetical events, without taking the nearest locations into consideration, the recognition of the spatial attribute of the event in the hypothetical class was significantly reduced in all the models trained by using the three techniques.

## Discussion

We conducted a detailed data analysis in order to find the main sources of errors in spatial attribute recognition. Based on the analysis, we found out that the main causes of the errors can be grouped into 5 cases, which are:

Case 1: Incorrect event class prediction

Most errors in this group occurred when an event in the normal class was classified by the model as an information class or vice versa. A major subcategory of the information class is a generic event (e.g. world knowledge), which, in this work, we assigned "world" as the spatial attribute. When a normal event was miss-classified as information, we often found that "world" was incorrectly recognized as the spatial attribute of the event.

Case 2: Error propagation

Errors sometimes occurred when the most reliable source for spatial attribute annotation of the current event relies on the predicted locations of another event, such as a previous event location, event coreference's location or a subject coreference's location that was identified according to the model-predicted location. For example, if the spatial attribute of the previous event, e_i-1_, was incorrectly recognized, then the spatial attribute of the event e_i _that relies on e_i-1 _will also be incorrectly recognized.

Case 3: Confusion between selecting the country-level and city-level nearest location

There is usually confusion, even for a human annotator, in deciding between selecting the location at the country-level or the lower level of administration as a spatial attribute of events. For example:

*The villages, some 10 km north Jowhar lack Health Care centers and children have been dying from contagious diseases for the last years as the official confirmed*.

The above example is an excerpt from an outbreak report for villages around Somalia's middle Shabele region. While the automatic system selected Jowhar as the spatial attribute, according to our annotation guidelines, the preference should be for the Shabelle region to be selected for the event "confirmed".

Base on the investigation, this type of error occurred most often with the reporting events.

Case 4: Spatial attribute recognition for information class

The information class can be subcategorized into generic knowledge and non-eventive clauses (for example, "The patient is a 6-year-old boy."). While the spatial attribute of generic knowledge is usually annotated as world or sometimes as a country-level location, the spatial attribute of a non-eventive clause is generally a specific location. *For example:*

*It is the first time something like this is happening at our school*.

The non-eventive information represented by the phrase "is the first time ..." should be anchored to the location of the school. Or,

*Local Community Health Care in the area told AFP that lack of health care is the main cause of the amazing children death number*.

The clause "*lack of health care is the main cause *..." refers to the fact in a specific region, (which, in this news, is "Shabele"), so Shabele was intuitively selected by the human annotator as the location of this non-eventive clause.

It is quite difficult to find explicit textual signals that can guide a model to select the most appropriate feature source for spatial attribute annotation. However, we often found that, in cases where the event is classified as information, when the subject of the verb referred to a non-specific, concept-level entity, such as "Bird flu", "Patients", "Children", the event represented by that verb is usually considered world knowledge, i.e. the spatial attribute is "World". Based on this observation, the analysis of a subject whether it refers to a concept level entity or not might help solve this error.

Case 5: Event that causes the spatial movement of the object

When the event involves the movement of an object from one place to another, both the source and destination locations should be recognized as the spatial attribute of the event. For example:

*There are fears the disease could spread into neighbouring Uganda*.

This news reported an outbreak in Sudan, so the hypothetical event "could spread" should be associated with a source location, e.g. Sudan, and the destination location, e.g. Uganda.

However, this type of event seldom occurred in our training corpus. So, it is difficult for the model to recognize this.

## Conclusions

In this article, we extended our work on event-spatial attribute recognition by focusing on machine learning techniques, which are CRF, SVM, and Decision tree. Our approach avoided the costly development of an external knowledge base by employing the feature sources that can be acquired locally from the analyzed document. The features incorporated to our models were evaluated to assess their contribution to the event-spatial attribute recognition task. The results showed that the CRF model performed the best. Moreover, apart from location-related features, subject type and event class are also important to the spatial attribute annotation. Among location-related features, our study indicated that the nearest location name and previous event location are the most important features for the CRF and SVM model, while the subject location feature is the most important to the Decision tree model. In the future, we plan to extend our experiment by selecting the best combination of the classifiers and features for each type of event.

In the experiment, the named entity, co-reference information as well as subjects and objects of all verbs were manually identified. In practice, such information can be automatically acquired by using linguistic tools, such as named entity annotation [21], parser [22] and co-reference resolution [27]. Since the errors from these tools could degrade the annotation accuracy, it is necessary to have the tools with acceptable performance. With the continual advances in natural language processing, we believe that such reliable tools can be obtained, and consequently, make the spatial attribute annotation reliable enough to be useful for practical purposes in online health surveillance systems.

In this study, we focused on analyzing outbreak reports in news articles. However, our methodology was designed to be independent of the specific patterns related to the language, document genre, or writer's idiosyncrasy. Therefore, we believe that it can be applied to other types of outbreak reports, such as ProMED mail, blog entries, or official reports.

## Competing interests

The authors declare that they have no competing interests.

## Authors' contributions

This work was directed by NC. HC carried out the framework design and analysis with the technical support and advice from NC. HC also carried out the experiments. Both authors contributed to the framework development and in writing the paper. Both authors read and approved the final manuscript.
